# Echinomycin as a promising therapeutic agent against KSHV-related malignancies

**DOI:** 10.1186/s13045-023-01441-5

**Published:** 2023-05-04

**Authors:** Jungang Chen, Zhen Lin, Jiao Song, Karlie Plaisance-Bonstaff, Jennifer James, Shengyu Mu, Steven R. Post, Lu Dai, Zhiqiang Qin

**Affiliations:** 1 Department of Pathology, Winthrop P. Rockefeller Cancer Institute, University of Arkansas for Medical Sciences, 4301 W Markham St, Little Rock, AR 72205 USA; 2 Department of Pathology, Tulane Cancer Center, Tulane University Health Sciences Center, New Orleans, LA 70112 USA; 3 Department of Medicine, Louisiana Cancer Research Center, Louisiana State University Health Sciences Center, New Orleans, LA 70112 USA; 4 Department of Pharmacology and Toxicology, University of Arkansas for Medical Sciences, Little Rock, AR 72205 USA

**Keywords:** KSHV, HHV-8, Myc, HIF1α, Echinomycin

## Abstract

Kaposi’s sarcoma-associated herpesvirus (KSHV) is the etiologic agent of several human cancers, including Kaposi’s sarcoma (KS) and primary effusion lymphoma (PEL), which preferentially arise in immunocompromised patients while lack of effective therapeutic options. Oncoproteins Myc and hypoxia-inducible factor-1α (HIF1α) have been found closely related to KSHV infection, replication and oncogenesis. However, the strategies of dual targeting these two oncoproteins have never been developed and tested for treatments of KSHV-related malignancies. In the current study, we report that treatment of echinomycin dramatically regresses cell growth both in vitro-cultured KSHV + tumor cells and in vivo KS or PEL xenograft mice models, through simultaneously inhibiting Myc and HIF1α expression. Echinomycin treatment also induces viral lytic gene expression whereas not increasing infectious virions production from KSHV + tumor cells. Our comparative transcriptomic analysis has identified a bunch of new Echinomycin-regulated, Myc- and HIF1α-related genes contributed to KSHV pathogenesis, including KDM4B and Tau, which are required for the survival of KSHV + tumor cells with functional validation. These data together reveal that dual targeting Myc and HIF1α such as using Echinomycin may represent a new and promising option for treatments of these virus-associated malignancies.

**Supplementary Information:**

The online version contains supplementary material available at 10.1186/s13045-023-01441-5.

**To the editor:**

Kaposi’s Sarcoma-associated Herpesvirus (KSHV) represents a principal causative agent of several cancers arising in patients with compromised immune systems, including Kaposi's Sarcoma (KS) and Primary Effusion Lymphoma (PEL) [1]. KSHV-induced malignancies represent a serious threat to immunosuppressed patients due to lack of effective therapies [2]. Myc is one of the most potent and commonly activated oncoproteins, whose activation is thus considered as a hallmark of cancer initiation and maintenance [3]. Hypoxia-inducible factor-1 (HIF1) is a master regulator mediating response to hypoxic stress in both normal tissues and tumors [4]. Echinomycin is a bis-intercalator peptide and is biosynthesized by a unique nonribosomal peptide synthetase (NRPS), and it belongs to a family of quinoxaline antibiotics. Interestingly, Huang et al. recently reported that Echinomycin simultaneously inhibited Myc and HIF1α through proteasomal degradation [5]. Although both Myc and HIF1α are found driven oncogenesis induced by KSHV [6, 7], dual targeting Myc and HIF1α by one agent against KSHV-related malignancies have never been reported.

Here we found that even at very low concentrations Echinomycin treatment effectively inhibited the growth of KSHV + tumor cell lines (CC_50_ only ~ 0.1–2 nM, Fig. 1A, B). In contrast, Echinomycin showed much less effective on the growth of normal cells such as HUVEC and peripheral blood B cells (CC_50_ >  > 1000 nM). In addition, Echinomycin showed effective inhibition of the growth of a KSHV-infected lymphoma cell line, BJAB.219, but much less on its parental KSHV negative cell line, BJAB (Additional file 1: Fig. S1). Our further data showed a dose-dependent and time-dependent inhibition of cell growth by Echinomycin for both TIVE-LTC and BCBL-1 cell lines (Fig. 1C, D). By using soft agar assays, we found that Echinomycin treatment effectively inhibited the anchorage independent growth of KSHV + tumor cells (Fig. 1E). By using a KS-like nude mice model [8], we found that Echinomycin treatment significantly repressed tumor growth in mice when compared to the vehicle-treated group (Fig. 1F). At the end of experiments, the tumors isolated from Echinomycin-treated mice shrunk with much smaller size than those from vehicle-treated mice (Fig. 1G). In addition, we found that Echinomycin treatment dramatically suppressed PEL tumor progression in an established xenograft model [9], including reducing ascites formation and spleen enlargement over this timeframe (Fig. 1H, I).

**Fig. 1:**
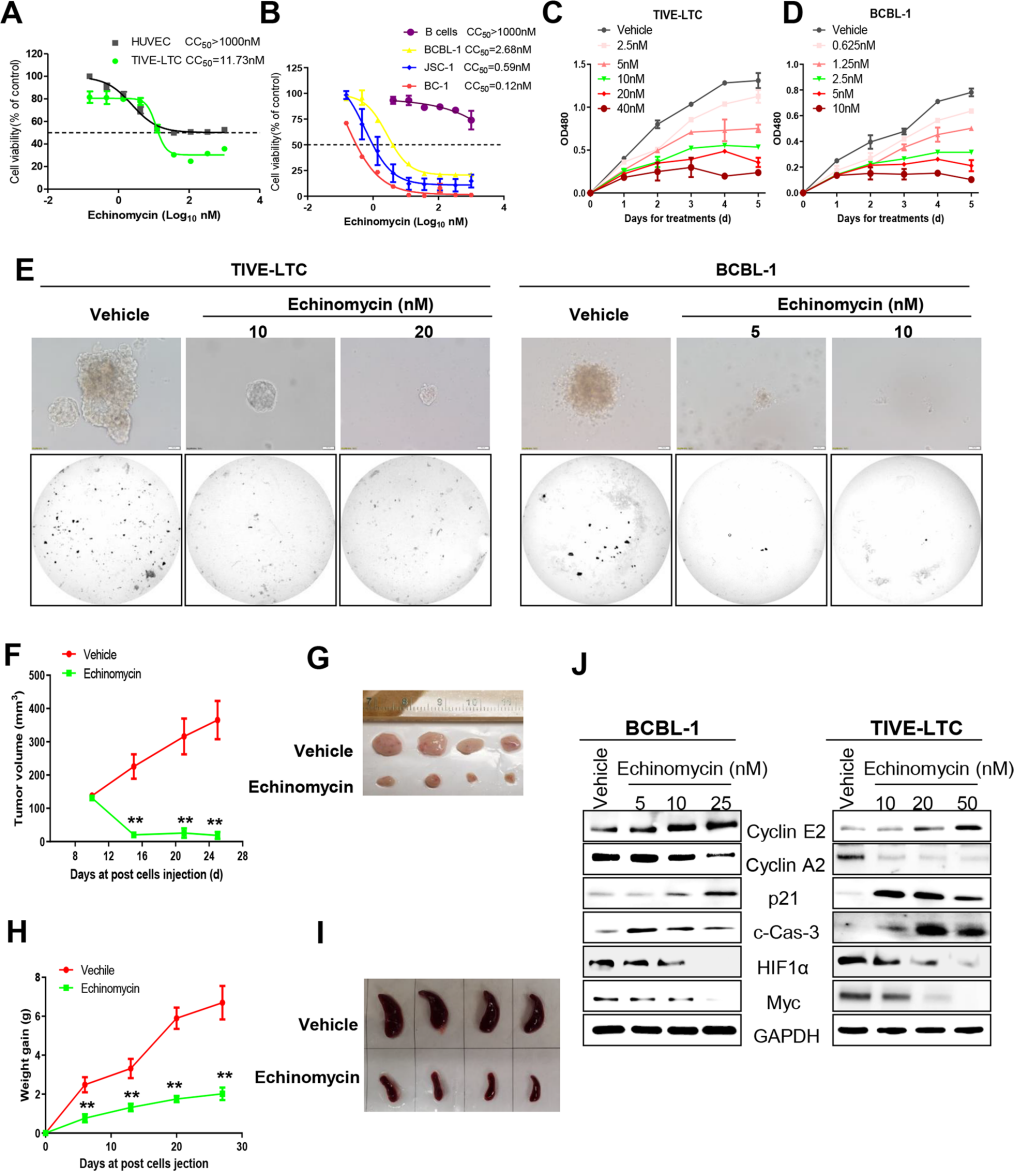
Echinomycin treatment inhibits the growth of KSHV + tumor cells in vitro and in vivo through repression of Myc and HIF1α. **A**–**D** Cells were treated with indicated concentrations of Echinomycin for a time-course, then cell viability was examined using the WST-1 proliferation assays (Roche). The 50% Cytotoxicity Concentrations (CC_50_) were calculated based on the dose-dependent “killing curves” by using GraphPad Prism v5.0. Error bars represent S.D. for 3 independent experiments. **E** The anchorage independent growth ability of TIVE-LTC and BCBL-1 were determined using the soft agar assays. **F**, **G** TIVE-LTC were injected subcutaneously into the flanks of nude mice. When tumors reach 10–15 mm in diameter, mice were randomly grouped and received in situ subcutaneous injection with either vehicle or Echinomycin (200 μg/kg). The mice were observed and measured every 4–5 days for the size of palpable tumors. At the end of experiment, the tumors were excised from the site of injection for comparison. **H**, **I** NOD/SCID mice were injected i.p. with BCBL-1 cells. 72 h later, the Echinomycin (2.5 μg/kg) or vehicle were administered i.p., and weights were recorded weekly. At the end of the treatment period, the spleens were collected for comparison. **p < 0.01. (**J**) BCBL-1 and TIVE-LTC were treated with indicated concentrations of Echinomycin for 48 h, then protein expression was measured by using Western blot

We further found that Echinomycin treatment significantly induced both BCBL-1 and TIVE-LTC cell apoptosis as well as cell cycle arrest (Additional file 1: Fig. S2). Echinomycin treatment affected the expression of several apoptosis- or cell cycle-related proteins through repression of Myc and HIF1α expression (Fig. 1J). Since Echinomycin has been found to promote Myc and HIF1α proteasomal degradation [5], our results confirmed that MG132 effectively prevented the reduction of Myc and HIF1α by Echinomycin from KSHV + tumor cells (Additional file 1: Fig. S3). Echinomycin treatment significantly increased the transcription and expression of viral lytic genes, such as RTA and ORF26 (Additional file 1: Fig. S4). However, in contrast to NaB (a classical lytic inducer) leading to a pronounced increase in mature virion production, Echinomycin displayed inhibitory effects on virion production from BCBL-1 cells, instead (Additional file 1: Fig. S4).

We then compared the gene profiles between vehicle- and Echinomycin-treated KSHV + tumor cell lines, using RNA-Sequencing analyses. The volcano plots showed the scattering of genes which were significantly upregulated or downregulated (FDR < 0.05) in Echinomycin-treated BCBL-1 or TIVE-LTC (Fig. 2A). The intersection analysis identified 234 genes commonly changed in both BCBL-1 and TIVE-LTC (Fig. 2B). The top 20 commonly upregulated or downregulated genes in both BCBL-1 and TIVE-LTC were listed in a heat map (Additional file 1: Fig. S5) as well as Additional file 1: Table S1. The GO_enrichment analysis of these commonly changed genes identified several major functional categories they belong to such as extracellular structure organization, regulation of apoptotic cells, nucleic acid metabolic process and regulation of humoral immune response (Additional file 1: Fig. S5).

**Fig. 2:**
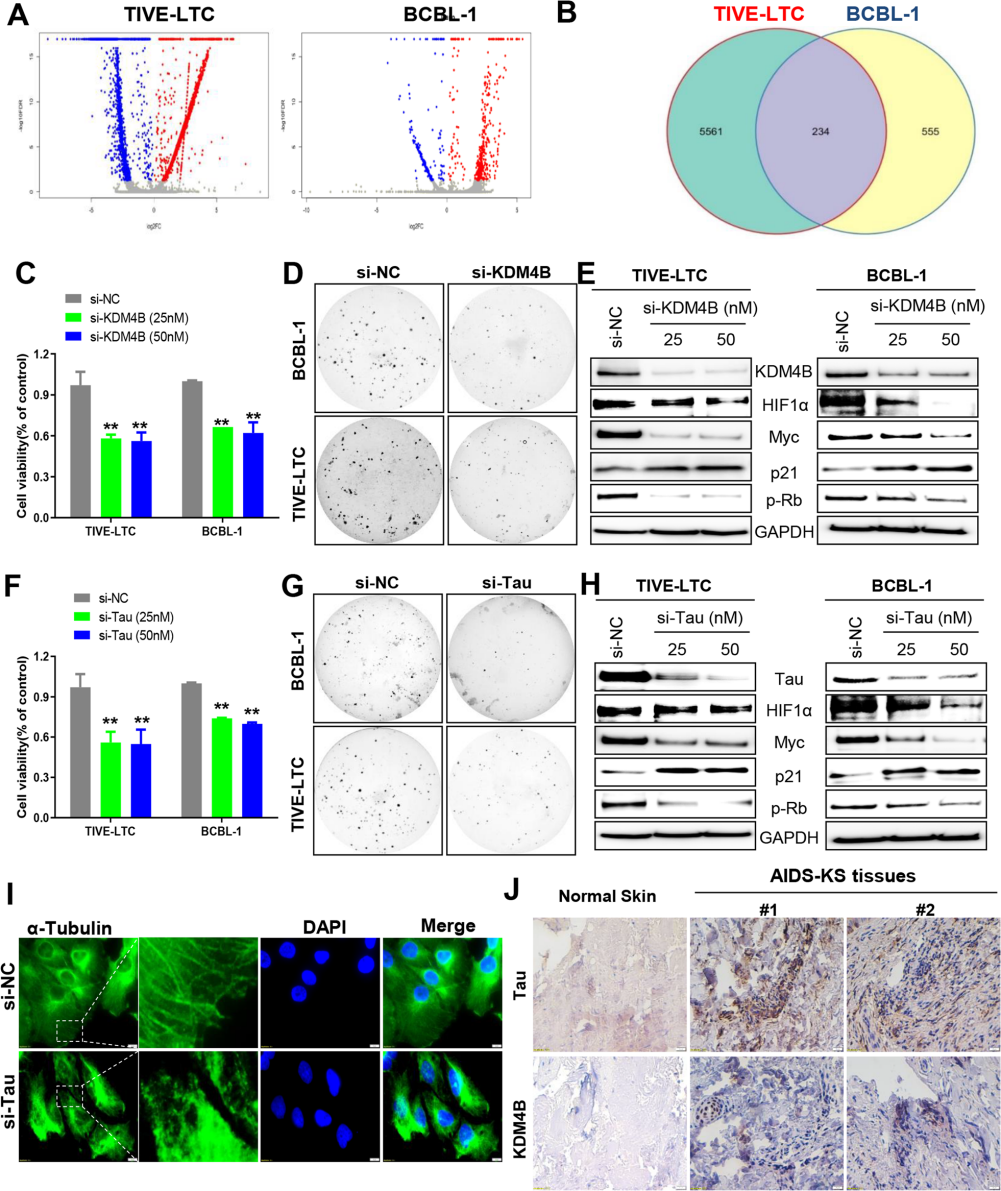
Identification of new Echinomycin-regulated genes which are contributed to KSHV pathogenesis. **A** RNA-Sequencing was used to investigate changes in the transcriptome between Echinomycin- and vehicle-treated TIVE-LTC and BCBL-1 cells. The significantly altered genes (*p* < 0.05) were shown in the Volcano plot panels. **B** The intersection analysis of unique and common genes altered in Echinomycin-treated TIVE-LTC and BCBL-1 cells. **C**–**H** TIVE-LTC and BCBL-1 cells were transfected with KDM4B-siRNA, Tau-siRNA or non-target control siRNA (si-NC) for 72 h, then cell proliferation, colony formation and protein expression were measured by using the WST-1 assays, soft agar assays and Western blot, respectively. Error bars represent S.D. for 3 independent experiments, **p < 0.01. **I** TIVE-LTC were transfected as described above, then microtubule formation was observed using immunofluorescence assays (IFA) with antibody targeting α-Tubulin. **J** The expression of KDM4B and Tau proteins in formalin-fixed paraffin-embedded KS tissues from cohort HIV + patients and normal skin tissues were measured and compared by using immunohistochemical (IHC) staining as described in the “Methods” section (the magnification at × 40)

We selected KDM4B (lysine demethylase 4B) and MAPT (microtubule associated protein Tau) for subsequent functional validation. KDM4B is broadly defined as an oncoprotein that plays key roles in processes related to tumorigenesis [10]. Tau is a protein that stabilizes and promotes the assembly of microtubules, which has been reported to be implicated in different types of cancer [11, 12]. We first confirmed the downregulation of these two proteins by Echinomycin in vitro and in vivo (Additional file 1: Fig. S6). Next, we demonstrated that direct knockdown of KDM4B or Tau by RNAi significantly inhibited the growth and colonies formation of KSHV + tumor cells (Fig. 2C, D, F, G), as well as downregulated the expression of both Myc and HIF1α (Fig. 2E, H). We further confirmed that direct knockdown of either Myc or HIF1α was able to downregulate both KDM4B and Tau expression from KSHV + tumor cells (Additional file 1: Fig. S7). By using immunofluorescence assay (IFA), knockdown of Tau severely impaired the structure and assembly of microtubules in TIVE-LTC (Fig. 2I). In addition, similar effects were observed within Echinomycin-treated TIVE-LTC in a dose-dependent manner (Additional file 1: Fig. S8). For clinical relevance, our results showed that the expression of KDM4B and Tau was upregulated in AIDS-KS tissues from two cancer patients when compared to normal skin tissues (Fig. 2J).

Taken together, our data reveal that dual targeting Myc and HIF1α by Echinomycin may represent a new and promising option for treatments of these virus-associated malignancies.

## Supplementary Information

**Additional file 1.** Supplementary methods, tables and figures.

## Data Availability

All the data shown in this paper are available from the corresponding authors upon reasonable request.

## Abbreviations

KSHV: Kaposi’s Sarcoma-associated Herpesvirus
KS: Kaposi’s Sarcoma
MCD: Multicentric Castleman Disease
PEL: Primary Effusion Lymphoma
HIF1α: Hypoxia-inducible factor-1α
Myc: Master regulator of cell cycle entry and proliferative metabolism
KDM4B: Lysine demethylase 4B
KDMs: The histone lysine demethylases
Tau/MAPT: Microtubule-associated protein
cART: Combination antiretroviral therapy
LANA: Latency-associated nuclear antigen
RTA: Replication and transcription activator
vGPCR: Viral G protein-coupled receptor
NGS: Next-generation sequencing analysis
RT-qPCR: Quantitative reverse transcription PCR
HUVEC: Human umbilical vein endothelial cells
TIVE-LTC: KSHV long-term-infected telomerase-immortalized HUVEC cells
CC_50_: The 50% cytotoxicity concentration
HO-1: Heme oxygenase-1
TS: Talaporfin sodium
PDT: Talaporfin sodium-photodynamic therapy
MIF: Migration inhibitory factor
ROS: Reactive oxygen species
SOD: Superoxide dismutase
