# Supplementary material for: Echinomycin as a promising therapeutic agent against KSHV-related malignancies
Source: J Hematol Oncol. 2023 May 4;16:48. doi: 10.1186/s13045-023-01441-5 (PMC10161613; doi:10.1186/s13045-023-01441-5)
Supplement: Supplementary file 1 — Additional file 1. Supplementary methods, tables and figures. [file 13045_2023_1441_MOESM1_ESM.docx]

**Supplementary Methods**

**Cell culture and reagents**

BCBL-1 were kindly provided by Dr. Pinghui Feng (University of Southern California). KSHV long-term-infected telomerase-immortalized HUVEC (TIVE-LTC) cells were kindly provided by Dr. Rolf Renne (University of Florida). BC-1, JSC-1, BJAB, HUVEC, HEK293T cells were purchased from American Type Culture Collection (ATCC), and cultured as recommended by the manufacturer. BJAB.219 cells were made as described previously (1). Human peripheral blood B cells were purchased from STEMCELL. Echinomycin was purchased from purchased from Selleck Chemicals.

**Cell proliferation and soft agar assays**

Cell proliferation was measured using the WST-1 Assay (Roche). Briefly, after the period of treatment of cells, 10 μL/well of cell proliferation reagent, WST-1 (4-[3-(4-Iodophenyl)-2-(4-nitro- phenyl)-2H-5-tetrazolio]-1,3-benzene disulfonate), was added into 96-well microplate and incubated for 3 h at 37°C in 5% CO_2_. The absorbance of samples was measured by using a microplate reader at 490 nm. The anchorage independent growth abilities were assessed using soft agar assays as described previously (2).

**Cell cycle and apoptosis analysis**

Flow cytometry was used for the quantitative assessment of cell cycle and apoptosis. Briefly, cell pellets were fixed in 70% ethanol, and incubated at 4°C overnight, then re-suspended in 0.5 mL of 0.05 mg/mL propidium iodide (PI) plus 0.2 mg/mL RNaseA, and incubated at 37°C for 30 min. Cell cycle distribution was analyzed using a BD Accuri C6 flow cytometer. Apoptosis was assessed by the FITC-Annexin V/PI Apoptosis Detection Kit I (BD Pharmingen) on a flow cytometer. Data was normalized as the fold change compared to the vehicle control.

**PEL and KS-like xenograft models**

For PEL xenograft model, NOD/SCID mice, 6–8-week old, male (Jackson Laboratory), 1 × 10^7^ BCBL-1 cells in 200 µL RMPI-1640 without FBS were injected intraperitoneally and then mice were randomized into treatment groups of 4 mice as described previously (3). Echinomycin or vehicle was administered initially at 72 h after BCBL-1 injections. Weights were recorded weekly as a surrogate measure of tumor progression. For KS-like xenograft models, 5 × 10^5^ TIVE-LTC cells in 50 μL PBS plus 50 μL growth factor-depleted Matrigel (BD Biosciences) were together injected subcutaneously into the flanks of nude mice, 6–8-week old, male (Jackson Laboratory), as described previously (4). When tumors reach 10-15 mm in diameter, mice were randomly grouped (4 mice per group) and received in situ subcutaneous injection with either vehicle or Echinomycin. All protocols were approved by the University of Arkansas for Medical Sciences Animal Care and Use Committee (No. 3960) in accordance with national guidelines.

**RT-qPCR**

Total RNA was isolated using the RNeasy Mini kit (Qiagen), and cDNA was synthesized using a SuperScript III First-Strand Synthesis SuperMix Kit (Invitrogen). Primers used for amplification of target genes were listed in Supplementary Table 2. Amplification was carried out using an iCycler IQ Real-Time PCR Detection System, and cycle threshold (Ct) values were tabulated in duplicate for each gene of interest in each experiment. “No template” (water) controls were used to ensure minimal background contamination. Using mean Ct values tabulated for each gene, and paired Ct values for *β-actin* as a loading control, fold changes for experimental groups relative to assigned controls were calculated using automated iQ5 2.0 software (Bio-rad).

**RNA-Sequencing and enrichment analysis**

RNA-Sequencing of triplicate samples was performed by BGI Americas Corporation using their unique DNBSEQ sequencing technology. The completed RNA-Sequencing data was submitted to NCBI Sequence Read Archive (SRA# PRJNA923030). Raw sequencing reads were analyzed using the RSEM software (version 1.3.0; human GRCh38 genome sequence and annotation) and gene expression was quantified as previously described (5). The EBSeq software was utilized to call differentially expressed genes that were statistically significant using a false discovery rate (FDR) less than 0.05. Differentially expressed genes between Echinomycin- and vehicle-treated tumor cells were used as input for the GO_enrichment analyses.

**RNA interference (RNAi)**

For RNAi assays, HIF1α, Myc, KDM4B or Tau On-Target plus SMARTpool small interfering RNA (siRNA; Dharmacon) or negative control siRNA were delivered using the DharmaFECT transfection reagent as recommended by the manufacturer.

**Western blot**

Total cell lysates (20 µg) were resolved by 10% SDS-PAGE, transferred to nitrocellulose membranes, and immunoblotted with antibodies to HIF1α (GeneTex), p21, LANA (Abcam), Myc, p-Rb, Cyclin A2, Cyclin E2, cleaved Capsase-3, cleaved PARP, KDM4B, Tau and GAPDH (Cell Signaling). The antibodies for viral proteins RTA and ORF26 were purchased from Helmholtz Munich, Germany. Immunoreactive bands were identified using an enhanced chemiluminescence reaction (Perkin-Elmer) and visualized by autoradiography.

**AIDS-KS tumor tissues**

KS tissues from patients with HIV infection and normal skin tissues were provided by the Louisiana State University Health Sciences Center (LSUHSC) HIV Outpatient (HOP) Clinic and Biospecimens Bank.

**Immunohistochemistry**

Formalin-fixed, paraffin-embedded tissues were microtome-sectioned to a thickness of 4 µm, placed on electromagnetically charged slides (Fisher Scientific). Immunohistochemistry was performed as described previously (6). The antibodies for KDM4B and Tau were purchased from Cell Signaling and used as recommended by the manufacturer. Images were collected using an Olympus BX61 microscope equipped with a high resolution DP72 camera and CellSense image capture software.

**Immunofluorescence assays**

Cells were seeded in eight-well chamber slides (Nunc) for different treatments, then fixed with 4% PFA and stained with a mouse Anti-tubulin monoclonal antibody (Sigma), followed by a goat anti-mouse secondary antibody conjugated to 488 (Invitrogen) and DAPI. Fluorescence signal was measured using the Olympus IX83 microscope (Olympus).

**Measurement of infectious virions production**

The infectious virions production was determined by using the infectivity assay as described previously (1). Briefly, the supernatant from the treated cells were collected to infect naïve HEK293T cells by spinoculation with the centrifugation at 1500×g for 60 min, then the viral DNA levels in HEK293T cells were quantified by qPCR assay with LANA specific primers at 24 h post-infection.

**Statistical analysis**

Significant differences between experimental and control groups were determined using the two-tailed Student’s *t*-test, and *p* < 0.05 or 0.01 were considered significant or highly significant, respectively. The 50% Cytotoxicity Concentrations (CC_50_) were calculated by using GraphPad Prism v5.0.

**Reference**

1. Chen J, Wang Z, Phuc T, Xu Z, Yang D, Chen Z*, et al.* Oncolytic strategy using new bifunctional HDACs/BRD4 inhibitors against virus-associated lymphomas. PLoS Pathog **2023**;19:e1011089

2. Dai L, Del Valle L, Miley W, Whitby D, Ochoa AC, Flemington EK*, et al.* Transactivation of human endogenous retrovirus K (HERV-K) by KSHV promotes Kaposi's sarcoma development. Oncogene **2018**;37:4534-45

3. Dai L, Trillo-Tinoco J, Cao Y, Bonstaff K, Doyle L, Del Valle L*, et al.* Targeting HGF/c-MET induces cell cycle arrest, DNA damage, and apoptosis for primary effusion lymphoma. Blood **2015**;126:2821-31

4. Dai L, Qiao J, Nguyen D, Struckhoff AP, Doyle L, Bonstaff K*, et al.* Role of heme oxygenase-1 in the pathogenesis and tumorigenicity of Kaposi's sarcoma-associated herpesvirus. Oncotarget **2016**;7:10459-71

5. Kheir F, Zhao M, Strong MJ, Yu Y, Nanbo A, Flemington EK*, et al.* Detection of Epstein-Barr Virus Infection in Non-Small Cell Lung Cancer. Cancers **2019**;11

6. Qin Z, Freitas E, Sullivan R, Mohan S, Bacelieri R, Branch D*, et al.* Upregulation of xCT by KSHV-encoded microRNAs facilitates KSHV dissemination and persistence in an environment of oxidative stress. PLoS Pathog **2010**;6:e1000742

**Supplementary Table 1. The top 20 genes upregulated or downregulated in Echinomycin-treated KSHV+ tumor cells.**

| **Gene_ID** | **Gene description** | **Fold changes** | |
| --- | --- | --- | --- |
|  |  | **BCBL-1** | **TIVE-LTC** |
| AMIGO3 | adhesion molecule with Ig like domain 3 | 35.32136 | 36.91974 |
| GRHL3 | grainyhead like transcription factor 3 | 11.9379 | 35.48236 |
| TMED7-TICAM2 | TMED7-TICAM2 readthrough | 41.83945 | 2.64259 |
| RN7SK | RNA component of 7SK nuclear ribonucleoprotein | 5.284894 | 23.82485 |
| BTG2 | BTG anti-proliferation factor 2 | 6.870981 | 16.37303 |
| C4A | complement C4A (Rodgers blood group) | 12.63843 | 6.890993 |
| PIK3R2 | phosphoinositide-3-kinase regulatory subunit 2 | 13.08326 | 6.071242 |
| EDA2R | ectodysplasin A2 receptor | 16.47162 | 1.978319 |
| COL11A2 | collagen type XI alpha 2 chain | 12.80776 | 5.564623 |
| CYP21A1P | cytochrome P450 family 21 subfamily A member 1, pseudogene | 10.92308 | 5.315432 |
| C4B | complement C4B (Chido blood group) | 11.33593 | 4.352721 |
| CYP21A2 | cytochrome P450 family 21 subfamily A member 2 | 11.18536 | 4.459285 |
| RASGRP3 | RAS guanyl releasing protein 3 | 13.12607 | 2.462569 |
| TAP2 | transporter 2, ATP binding cassette subfamily B member | 13.40053 | 1.815987 |
| GADD45B | growth arrest and DNA damage inducible beta | 6.626093 | 7.827677 |
| IRF7 | interferon regulatory factor 7 | 8.228955 | 4.932223 |
| TECTA | tectorin alpha | 4.379253 | 8.367941 |
| IL6 | interleukin 6 | 10.08992 | 2.604457 |
| NAP1L5 | nucleosome assembly protein 1 like 5 | 4.741842 | 6.497289 |
| HSPA1L | heat shock protein family A (Hsp70) member 1 like | 4.650578 | 6.572215 |
| TRPM4 | transient receptor potential cation channel subfamily M member 4 | 0.409312 | 0.147859 |
| CDK18 | cyclin dependent kinase 18 | 0.449136 | 0.102376 |
| CUX1 | cut like homeobox 1 | 0.35565 | 0.192502 |
| MID1 | midline 1 | 0.389154 | 0.156709 |
| CLIP2 | CAP-Gly domain containing linker protein 2 | 0.38584 | 0.157618 |
| WWOX | WW domain containing oxidoreductase | 0.353629 | 0.167924 |
| ANKS1B | ankyrin repeat and sterile alpha motif domain containing 1B | 0.470917 | 0.039069 |
| THSD4 | thrombospondin type 1 domain containing 4 | 0.349921 | 0.158805 |
| TBC1D22A | TBC1 domain family member 22A | 0.46156 | 0.044264 |
| SASH1 | SAM and SH3 domain containing 1 | 0.307826 | 0.197553 |
| TRAPPC9 | trafficking protein particle complex subunit 9 | 0.435921 | 0.067619 |
| LAMB1 | laminin subunit beta 1 | 0.45483 | 0.043663 |
| CILP | cartilage intermediate layer protein | 0.35758 | 0.126021 |
| MAPT | microtubule associated protein tau | 0.411854 | 0.071247 |
| ARL4C | ADP ribosylation factor like GTPase 4C | 0.447004 | 0.033153 |
| SEMA6B | semaphorin 6B | 0.356438 | 0.10724 |
| MAD1L1 | mitotic arrest deficient 1 like 1 | 0.34079 | 0.111205 |
| SLC29A4 | solute carrier family 29 member 4 | 0.311165 | 0.129147 |
| KDM4B | lysine demethylase 4B | 0.351115 | 0.075195 |
| AGAP1 | ArfGAP with GTPase domain, ankyrin repeat and PH domain 1 | 0.314681 | 0.056114 |

**Supplementary Table 2. Primer sequences for RT-qPCR in this study.**

| **Gene** | **Forward primers** | **Reverse primers** |
| --- | --- | --- |
| LANA | 5'-TCCCTCTACACTAAACCCAATA-3' | 5'-TTGCTAATCTCGTTGTCCC-3' |
| RTA | 5’-CACAAAAATGGCGCAAGATGA-3’ | 5’-TGGTAGAGTTGGGCCTTCAGTT-3’ |
| ORF26 | 5’-GCTCGAATCCAACGGATTTG -3’ | 5’- AATAGCGTGCCCCAGTTGC-3’ |
| β-actin | 5’-ATCGTGCGTGACATTAAGGAG-3’ | 5’-GGAAGGAAGGCTGGAAGAGT-3’ |

**Supplementary Figure Legends**

**Supplementary Figure 1. Echinomycin treatment selectively inhibits the growth of KSHV+ tumor cells.** BJAB and BJAB.219 cells were treated with indicated concentrations of Echinomycin for 48 h, then cell viability was examined using the WST-1 proliferation assays (Roche).

**Supplementary Figure 2. Echinomycin treatment induces apoptosis and cell cycle arrest from KSHV+ tumor cells.** (**A-D**) BCBL-1 and TIVE-LTC were treated with indicated concentrations of Echinomycin for 48 h, then cell apoptosis and cell cycle were measured by using Annexin V-PI staining and flow cytometry analysis. Error bars represent S.D. for 3 independent experiments, * = p<0.05, ** = p<0.01.

**Supplementary Figure 3. MG132 treatment protects Myc and HIF1α expression reduced by Echinomycin.** (**A**) TIVE-LTC and BCBL-1 cells were treated with Echinomycin in combination of MG132, then protein expression was measured by using Western blot. (**B**) Cells were treated with MG132 alone, then protein expression was measured by using Western blot.

**Supplementary Figure 4. Echinomycin treatment induces viral lytic gene expression but not infectious virions production from KSHV+ tumor cells.** (**A-B**) BCBL-1 were treated with indicated concentrations of Echinomycin for 48 h, then representative latent and lytic gene transcription and expression were measured by RT-qPCR and Western blot, respectively. (**C**) BCBL-1 cells were treated by indicated concentrations of NaB (a positive control), Echinomycin or vehicle, then the viral supernatants were collected to infect naïve HEK293T cells. The viral DNA levels were measured by using qPCR. Error bars represent S.D. for 3 independent experiments, * = p<0.05, ** = p<0.01.

**Supplementary Figure 5. Transcriptome analysis of Echinomycin-treated KSHV+ tumor cells.** (**A**) RNA-Sequencing was used to investigate changes in the transcriptome between Echinomycin- and vehicle-treated TIVE-LTC and BCBL-1 cells. The heat map of top 20 commonly and significantly upregulated or downregulated genes in Echinomycin-treated TIVE-LTC and BCBL-1 cells. (**B**) The GO_enrichment (Biological process) analysis of the commonly changed genes in Echinomycin-treated TIVE-LTC and BCBL-1 cells.

**Supplementary Figure 6. Echinomycin treatment reduced KDM4B and Tau expression from KSHV+ tumor cells *in vitro* and *in vivo*.** (**A**) Cells were treated by indicated concentrations of Echinomycin or vehicle for 48 h, then protein expression was measured by using Western blot. (**B**) The expression of KDM4B and Tau in tumor tissues collected from vehicle- or Echinomycin-treated KS-like xenograft mice were detected and compared using IHC staining.

**Supplementary Figure 7. Direct knockdown of Myc or HIF1α downregulates KDM4B and Tau expression from KSHV+ tumor cells.** Cells were transfected with Myc-siRNA, HIF1α-siRNA or non-target control siRNA (si-NC) for 72 h, then protein expression was measured by using Western blot.

**Supplementary Figure 8. Echinomycin treatment impaired microtubule functions in KSHV+ tumor cells.** TIVE-LTC were treated by indicated concentrations of Echinomycin, then microtubule formation was observed using immunofluorescence assays (IFA) with antibody targeting α-Tubulin.

**Supplementary Figure 1**


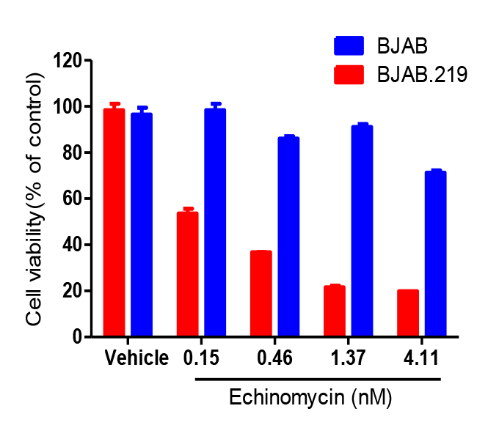


**Supplementary Figure 2**


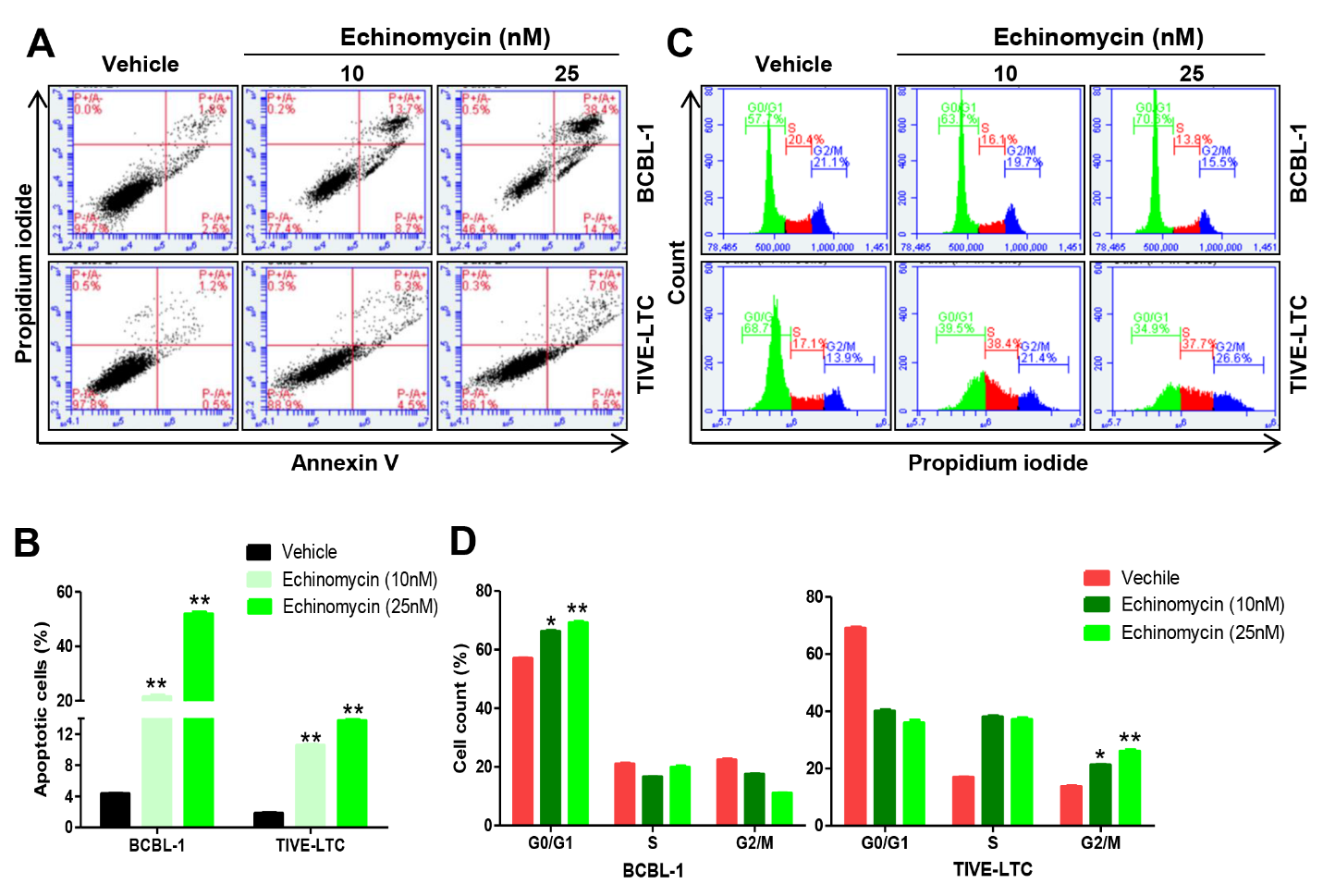


**Supplementary Figure 3**


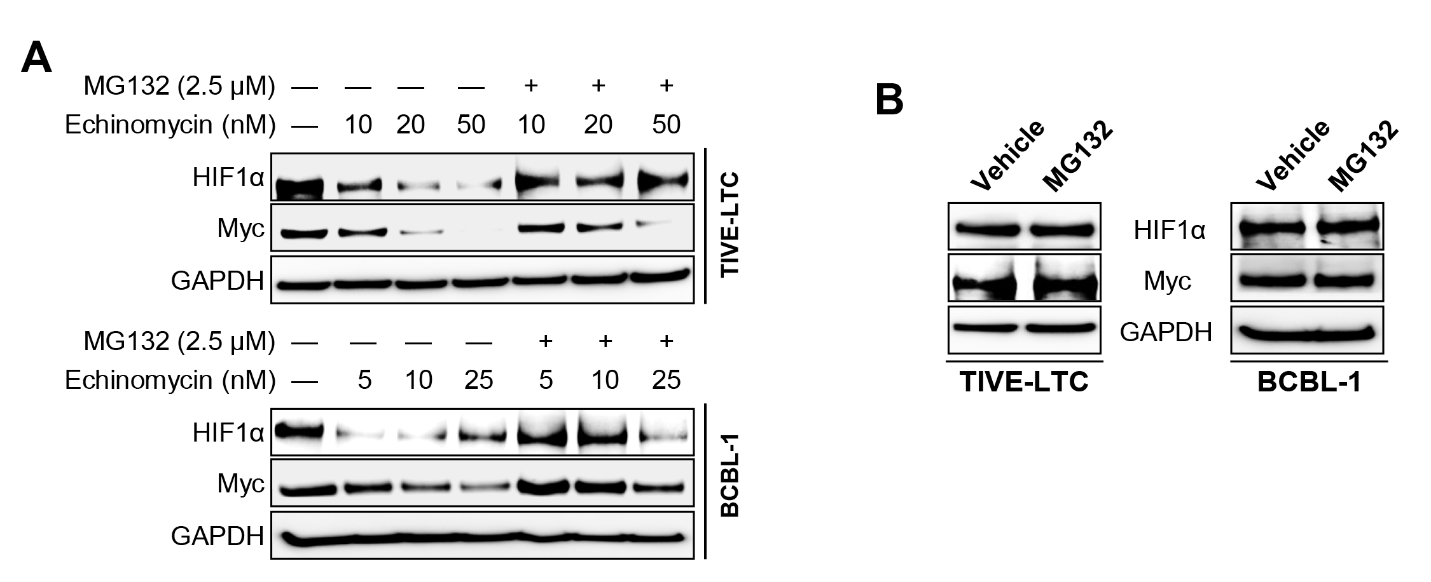


**Supplementary Figure 4**


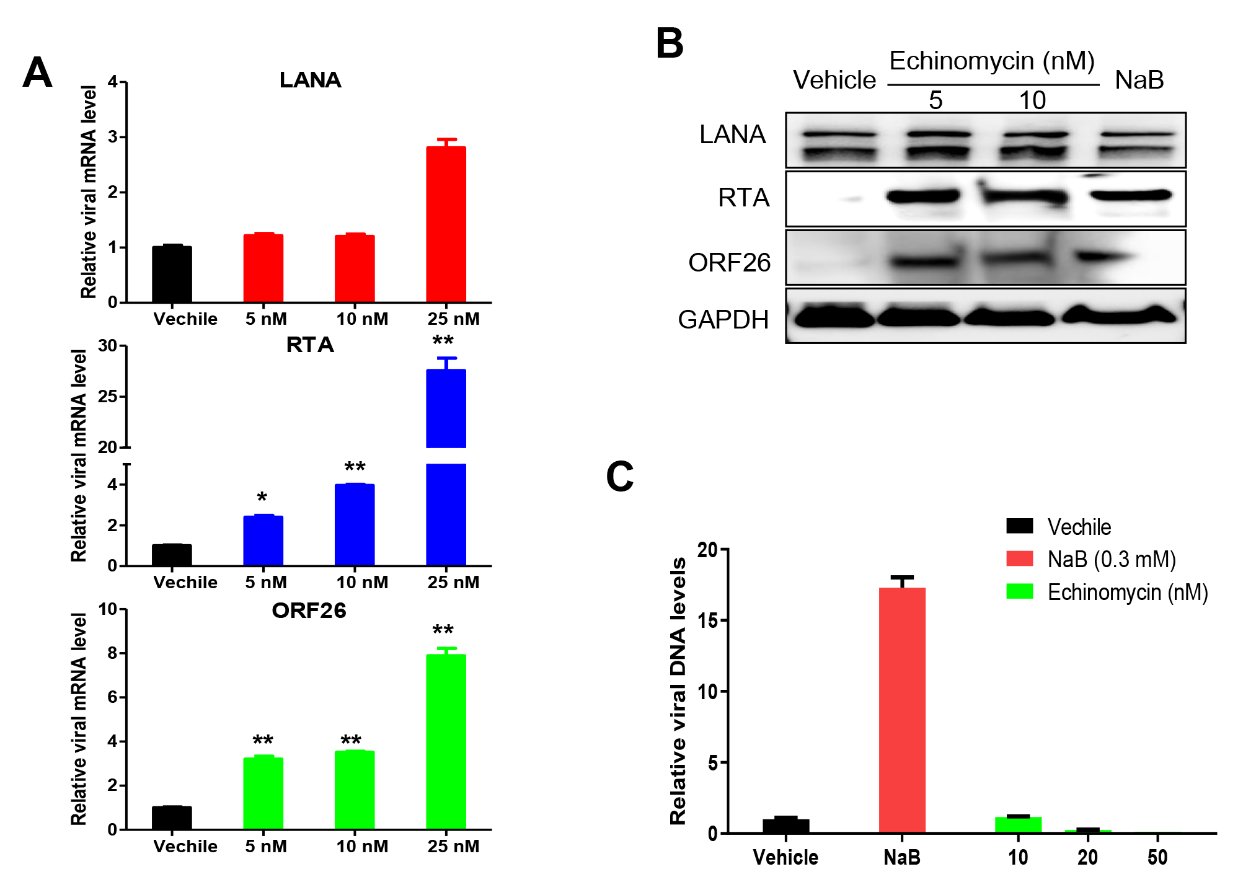


**Supplementary Figure 5**


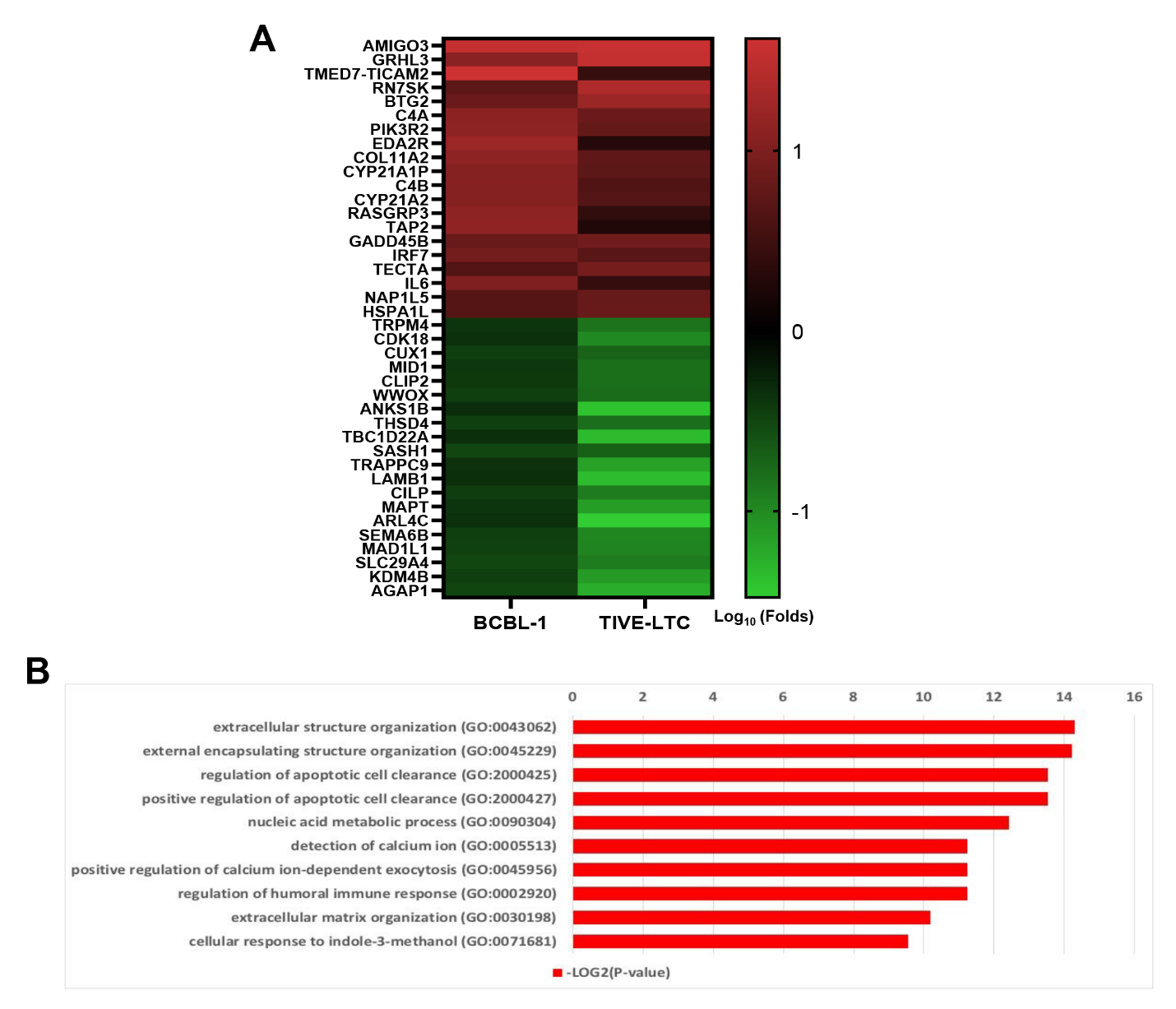


**Supplementary Figure 6**


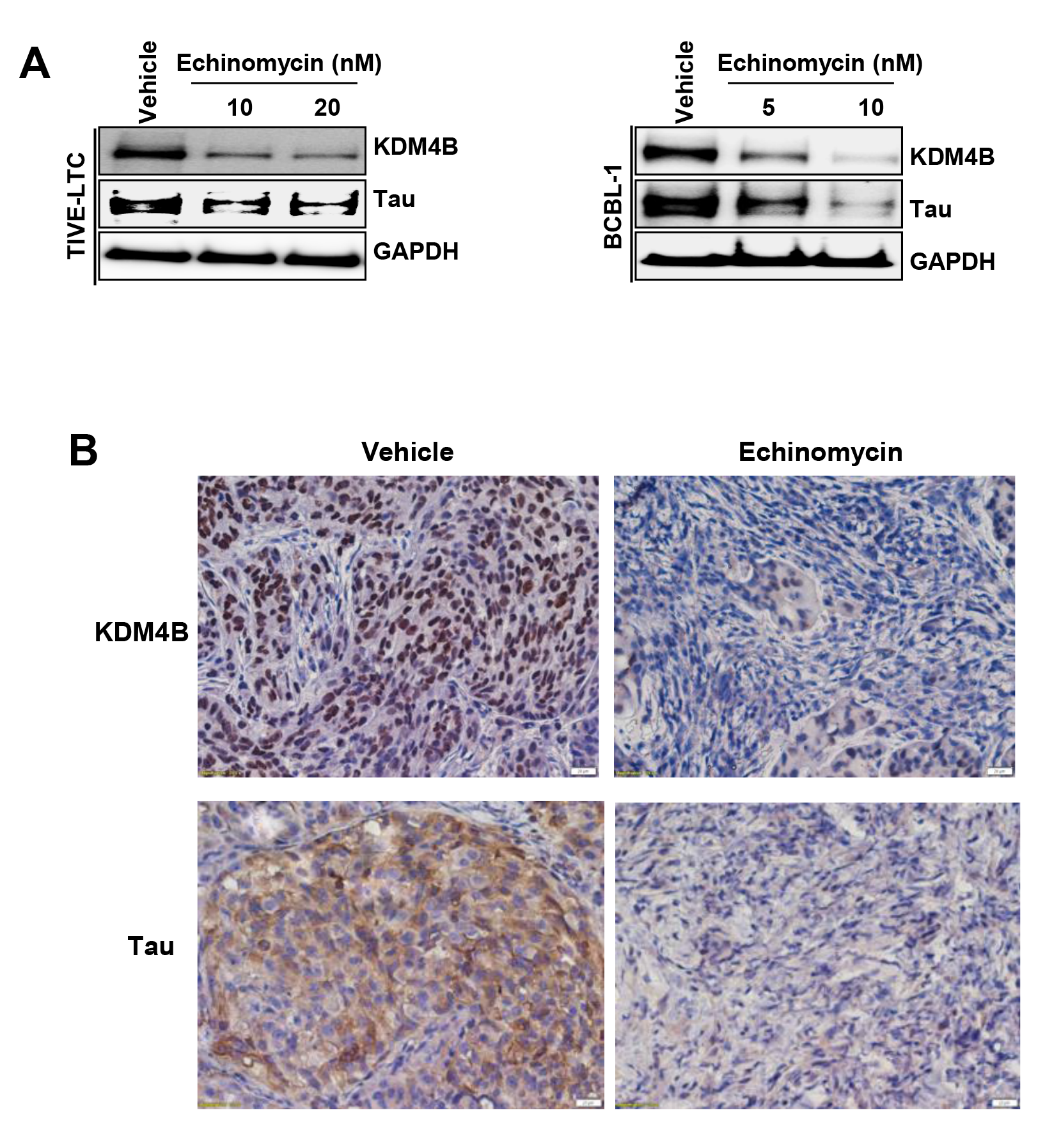


**Supplementary Figure 7**

**
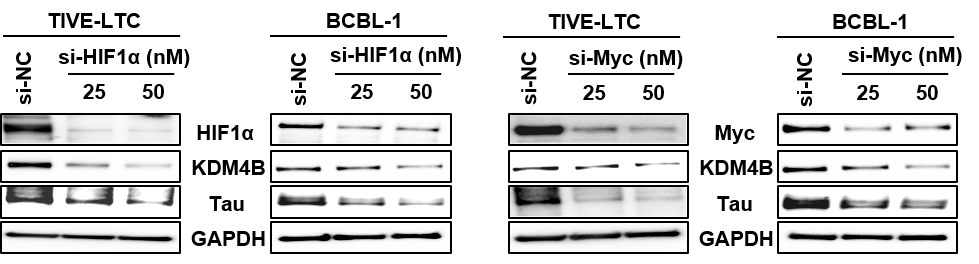
**

**Supplementary Figure 8**

**
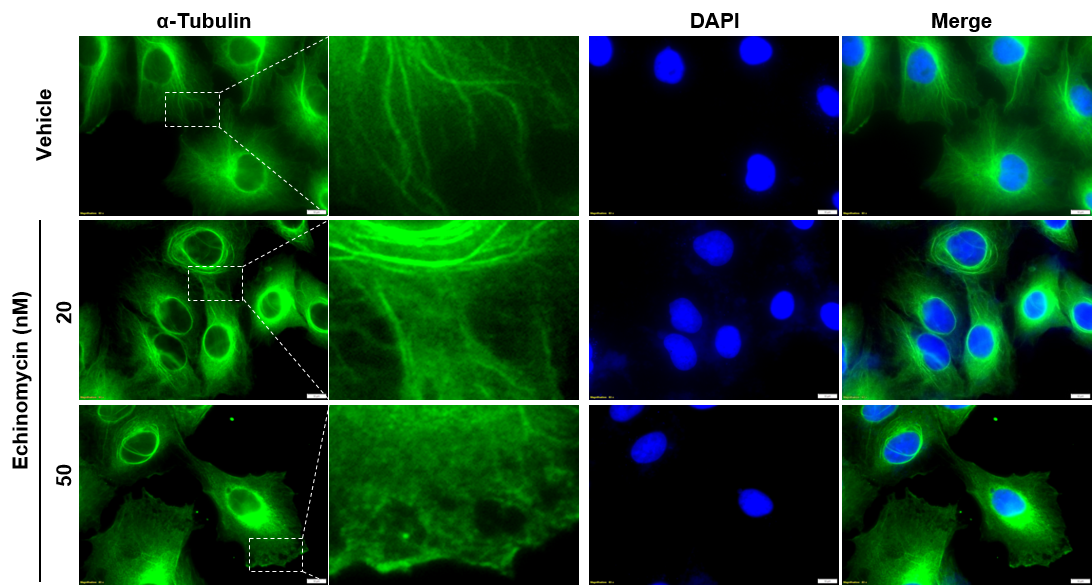
**
